# An Innovative Breast-Conserving Oncoplastic Technique for Treating Small to Medium Volume Breasts With a Tumor in the Lower Quadrant: The Folding Flap Technique

**DOI:** 10.3389/fonc.2022.841682

**Published:** 2022-03-04

**Authors:** Wenjie Shi, Maoli Wang, Luz Angela Torres-de la Roche, Xuefeng Shi, Zhenyu Gong, Jie Dong, Zihao Chen, Jiajia Zeng, Yicheng Jiang, Zhitong Chen, Kejin Wu, Rui Zhuo, Rudy Leon De Wilde

**Affiliations:** ^1^University Hospital for Gynecology, Pius-Hospital, University Medicine Oldenburg, Oldenburg, Germany; ^2^Department of Breast Surgery, Obstetrics and Gynecology Hospital of Fudan University, Shanghai, China; ^3^Department of Breast Surgery, EUSOMA Certified Breast Center, Guilin TCM Hospital of China, Guilin, China; ^4^International Center for Aesthetic Medicine, Affiliated Hospital of Guilin Medical University, Guilin, China

**Keywords:** breast cancer, oncoplastic, breast conserving, lower quadrant, folding flap

## Abstract

**Background:**

Here, we describe an innovative oncoplastic technique for small to medium volume breasts with a tumor in the lower quadrant and this technique could provide sufficient tissue to avoid visible defects after tumor removal and help reshape the natural shape of the breast.

**Methods:**

A detailed procedure for the folding flap technique is described step by step. Then, the results of a retrospective analysis of patients treated using this technique, including complications and disease recurrence rate, between January 2017 and November 2021 are reported. Aesthetic outcomes were evaluated on a 5-point scale proposed by the Paris Breast Center.

**Results:**

A total of 52 patients underwent surgery with the folding flap technique, The average operation time was 98.4 min (range, 75–120 min), and the mean bleeding volume was 56.5 mL (range, 20–100 mL). A margin-positive result was confirmed in 1 patient who underwent re-excision. Short-term postoperative complications were observed in 7 patients, including 4 with fat liquefaction, 2 with seroma, and 1 with skin redness and swelling. No flap necrosis was observed. The median follow-up time was 28.6 months (range, 9–58 months), and 2 patients experienced local recurrence. The mean aesthetic score was 4.7 points, with 36 patients scoring 5 points and 26 patients scoring 4 points, respectively.

**Conclusions:**

The folding flap technique, as an innovative and favorable oncoplastic technique for treating small- to medium-volume breasts with a tumor in the lower quadrant, could retain sufficient tissue to fill the residual cavity after the operation while improving the aesthetic outcome of the breast.

## Introduction

Breast-conserving surgery (BCS) is considered an effective treatment means to cure tumors while maintaining a relatively satisfying appearance (1). However, since tumors can be found in different quadrants, not all patients who undergo BCS experience satisfactory cosmetic outcomes after the procedure (2). Removal of a tumor located in the lower quadrant, compared to other quadrants, is more likely to result in postoperative breast deformity because, after tumor removal, the remaining glandular tissue is generally insufficient to completely fill the defect, affecting the symmetry and aesthetic appearance of the breast (3, 4). Especially, such deformities can occur in breast cancer patients with small- to medium-volume breasts (5). Therefore, performing safe oncological surgery that yields good aesthetic results is more challenging in these patients.

To overcome this obstacle, the breast oncoplastic (OPS) technique, as an alternative option, was proposed, providing a more satisfactory solution for the abovementioned patients. The core concept of this technique is the application of plastic surgery skills to oncological surgery, through cooperation between oncologists and plastic surgeons, to remove the tumor while providing a better appearance of the breast (6). At present, among all the OPS techniques, the volume-replacement technique, as an important part of the breast OPS procedure, is often used to repair glandular defects of the lower quadrant in small- to medium-volume breasts (7). When using the volume-replacement technique, surgeons collect tissues patches from other areas of the body to replace the missing mammary gland tissue. Although this can reshape the natural appearance of the breasts, some procedures will lead to severe postoperative complications and thereby reduce patient satisfaction (8, 9). Thus, the development of new and mature oncoplastic techniques, such as those providing more flexible pedicle flaps, is necessary to improve surgical outcomes while minimizing postoperative complications.

Here, we present the steps of an innovative and favorable folding flap technique for treating small- to medium-volume breasts with a tumor in the lower quadrant. We then report the findings of a retrospective analysis of surgical and aesthetic outcomes of patients who were treated using this technique.

## Materials and Methods

**Figure 1:**
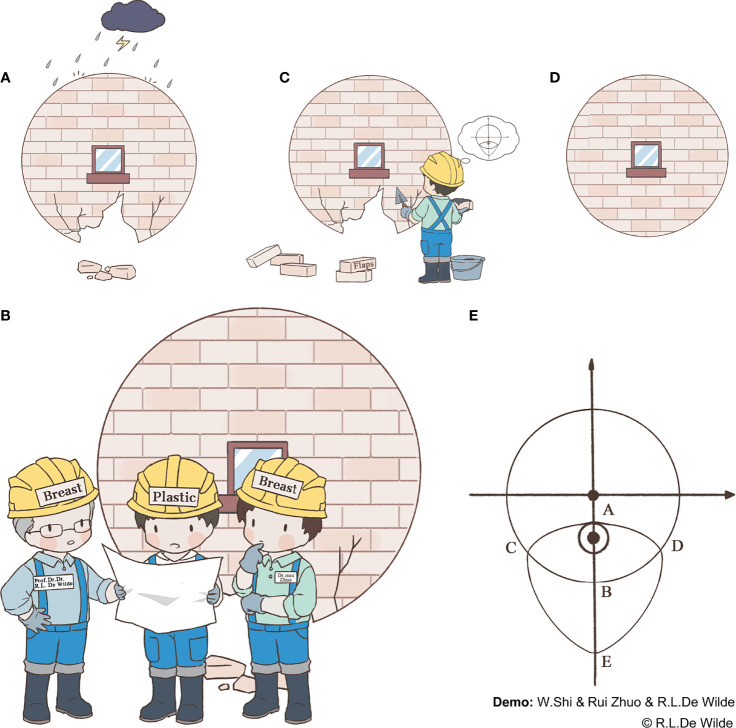
Workflow and approach design for the folding flap technique.

Glandular defects caused by damage from risk factors, such as a tumor or traumatic injury, affect the natural shape of the breast (**Figure 1A**). In such cases, a system repair plan needs to be discussed by the breast surgeon and plastic surgeon (**Figure 1B**). It is more difficult to repair defects in the lower quadrant of the breast, as the remaining tissue is often insufficient in this quadrant, especially in patients with small- to medium-volume breasts. As such, in this study, we propose a novel folding flap technique to solve this problem **(** **Figures 1C, D**). Patients who can be considered eligible candidates for this procedure include those with small- to medium-volume breasts with a tumor located in the lower quadrant, >2 cm away from the nipple, that accounts for 20%–50% of the total breast volume. A history of diabetes, smoking, breast surgery, or chest radiotherapy and the existence of multiple tumor lesions or a *BRCA* gene mutation are considered contraindications to this procedure. The folding flap technique in question is divided into several steps as follows.

### Incision Design and Tumor Resection

**Figure 2:**
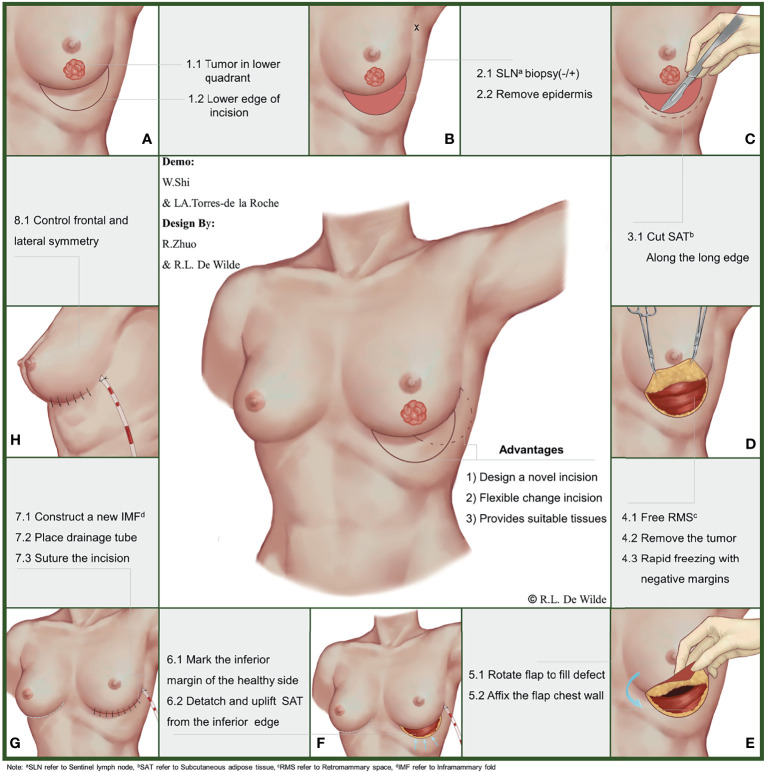
Demo figure for the folding flap technique.

During this stage of the procedure, the patient should be placed in a semi-recumbent position after general anesthesia and the tumor location marked on the body surface under ultrasound guidance. A circle should be drawn, using the tumor center on the body surface projection as a center point and an area 1 cm outside of the tumor border as the radius. Then, the nipple should be connected to the center of the circle and extended to the inframammary fold (IMF). The intersection of the line connecting with the circumference of the circle should be marked as point A, and the intersection with the IMF should be marked as point B. Point B should be taken as the center of the symmetry point E of point A. Arcs incorporating point A should be made on both sides, and the points where each arc intersects with the IMF should be noted as points C and D, respectively. Finally, the CDE arc should be drawn, completing the incision design of the tumor (**Figure 1E** and **Figure 2A**).

### Node Staging and De-Epithelialization

Importantly, all patients should undergo a routine surgical procedure for node staging, which should be completed *via* sentinel lymph node biopsy or axillary lymph node dissection. Patients with negative pathological results may be exempted from axillary lymph node dissection, but those with positive lymph node biopsies should undergo axillary lymph node dissection. De-epithelialization of the crescentic area should be completed in advance of preparing flap (**Figure 2B**).

### Tumor Resection

A cut into the skin should be made, along the arc-shaped incision, and the crescentic area along with part of the retro-mammary space should be freed completely. With support from an assistant, the operator should use a pulling hook to expose the operative area, then complete the excision of the tumor. The surgical margin can be evaluated on the frozen section during the operation to confirm complete removal of the tumor. Re-excision may be required to make sure that the surgical margin is negative (**Figures 2C, D**).

### Defect Repair and Aesthetic Evaluation

For flap preparation and defect filling, the operator should first check that the previous step was completed correctly. De-epithelialization of the donor flap is required to complete the preparation of the flap after the tumor removal. Then, the prepared flap may be folded along the IMF to fill the defect area while adjusting the fullness of the lower pole of the breast. After obtaining a satisfactory visual effect, the flap can be fixed to the pectoralis major muscle with a silk thread (**Figure 2E**).

In constructing the new IMF, methylene blue should be used to mark the IMF on the affected side with reference to the healthy side (**Figure 2F**). Then, the operator should free the lower edge of the subcutaneous adipose tissue and push it upward, along the arc-shaped incision. Once the subcutaneous adipose tissue overlaps with the marking point of the new IMF, it is then fixed to the chest, and a new IMF has been constructed successfully (**Figure 2G**).

### Evaluation of the Aesthetic Outcome and Follow-Up

An aesthetic evaluation should be performed by the surgical team immediately after surgery, focusing on breast shape, symmetry, and positioning of the nipple–areola complex (**Figure 2H**). Long-term observation of the aesthetic outcome may be carried out by 3 surgeons 6 months after the operation. The evaluation criteria for this are based on a 5-point scale (1 = poor aesthetic results, 5 = good aesthetic results) developed by the Paris Breast Center.

Patients who undergo this procedure should be followed up with regularly, and events such as postoperative complications, recurrence or metastasis of the tumor, and death should be registered.

### Application of the Technique to the Real World

**Figure 3:**
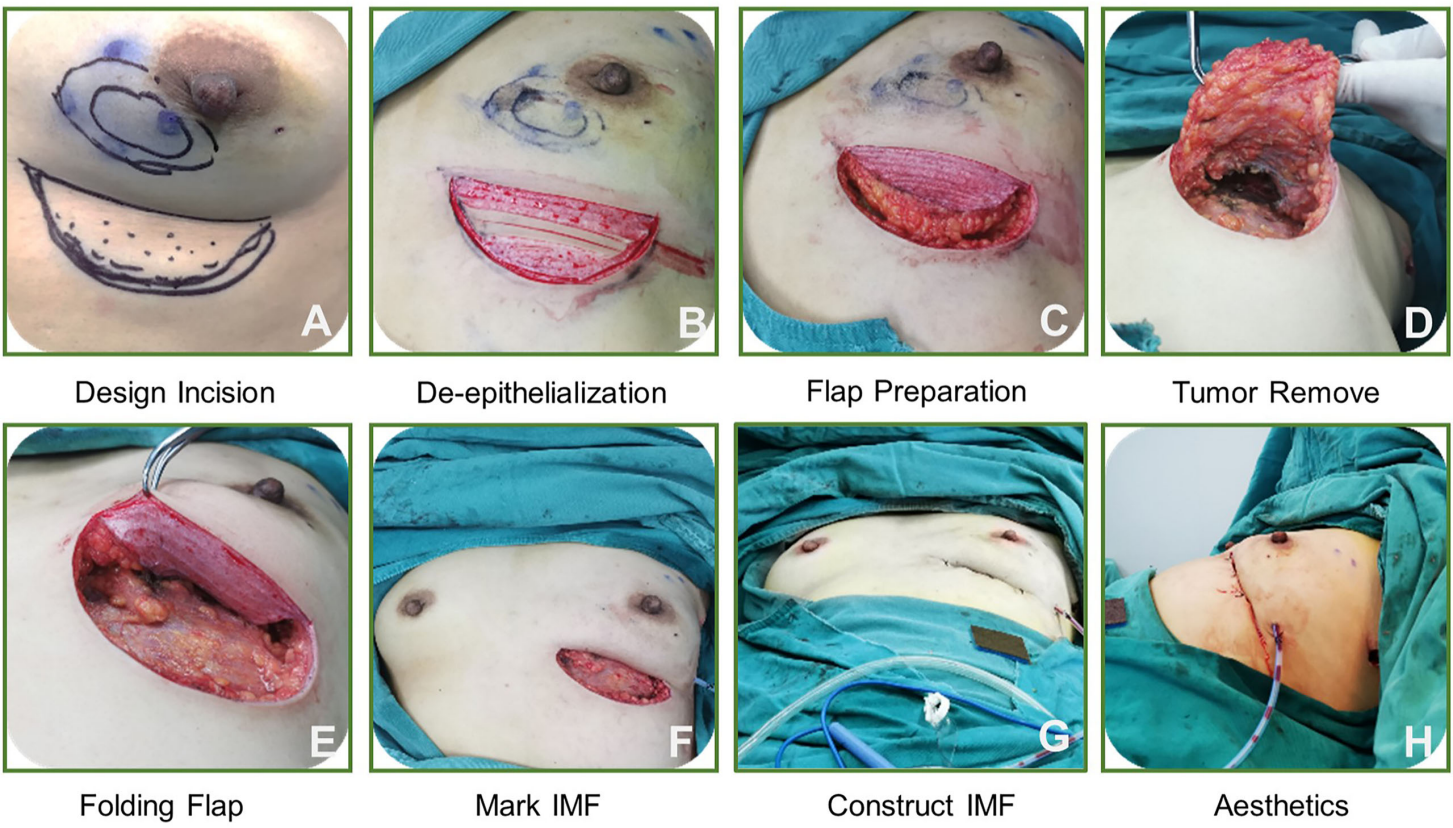
The step by step operation of a novel technique in clinical case.

We also discuss the operation of the folding flap technique in clinical cases (**Figure 3**), as follows. Overall, the technique includes 8 steps and the procedure is simple, making it easily masterable by surgeons.

## Results

### Baseline Characteristics of Enrolled Patients

Between January 2017 and November 2021, a total of 52 patients underwent surgery using the folding flap technique. The baseline information of these patients is displayed in **Table 1**. The percentage of patients aged <60 years was 86.5% (45/52). Patients with A-cup breasts (42.3%) and B-cup breasts (40.4%) were more common, while those with C-cup breasts accounted for 17.7% of the study population. Tumors measuring >2 cm were found in 65.4% of patients, and a Ki-67 concentration of >30% was confirmed in 22 patients. Among the enrolled patients, invasive ductal carcinoma was the most common pathological type (88.5%) and luminal B was the most common molecular type 50% (26/52) of breast cancer, respectively. One patient had mucinous adenocarcinoma. Tumors in the inner lower quadrant accounted for 44.2% (23/52) and tumors in the outer lower quadrant accounted for 32.7% (17/52) of all tumors, respectively.

**Table 1:** Baseline characteristic of enrolled patients.

Characteristic	Overall (N=52)	
	Number	%
**Age (Years)**		
≤60	45	86.5
>60	7	13.5
**BMI****^a^** **(Kg/m^2^)**		
18-25	38	73.1
>25	14	26.9
**Cup (%)**		
A	22	42.3
B	21	40.4
C	9	17.3
**Tumor Size (mm)**		
15-20	18	34.6
20-40	34	65.4
**Ki67 (%)**		
<15	11	21.2
15-30	19	36.5
>30	22	42.3
**Histology (%)**		
IDC^b^	46	88.5
ILC^c^	2	3.8
MA^d^	1	1.9
Other	3	5.8
**Subtype (%)**		
Luminal A	10	19.2
Luminal B	26	50.0
HER2	13	25.0
TNBC^e^	3	5.8
**Location (%)**		
Lower Half	12	23.1
Lower Inner	23	44.2
Lower Outer	17	32.7

a Body Mass Index

b Invasive ductal carcinoma.

c Invasive lobular carcinoma.

d Triple Negative Breast Cancer.

e Mucinous adenocarcinoma.

### Surgical Information and Complications

**Figure 4:**
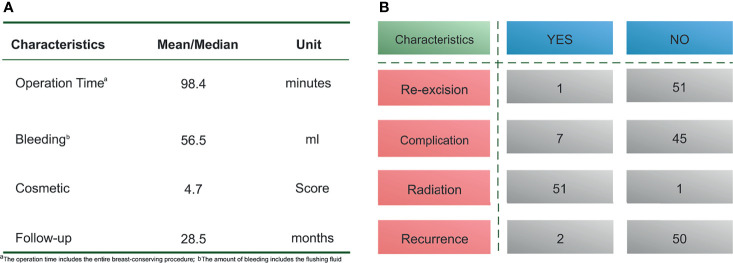
Surgical information and complications of patients.

The average operative time was 98.4 min (range, 75–120 min), and the mean bleeding volume was 56.5 mL (range, 20–100 mL). One patient was found to have positive margins by rapid freezing and underwent re-excision; their second pathology report subsequently confirmed margin negativity. Short-term postoperative complications were observed in 7 patients, including 4 with fat liquefaction, 2 with seroma, and 1 with localized skin redness and swelling. These complications did not lead to a delay in postoperative adjuvant therapy, and the follow-up results suggested that all folded flaps persisted without necrosis (**Figures 4A, B**).

### Tumor Safety and Cosmetic Outcome

The median follow-up time was 28.6 months (range, 9–58 months), and local recurrence occurred in 2 patients. All patients received chemotherapy. According to their molecular subtype, patients were also treated with endocrine therapy or targeted therapy. One patient refused to undergo postoperative radiotherapy because of concerns about the side effects of radiation therapy.

**Figure 5:**
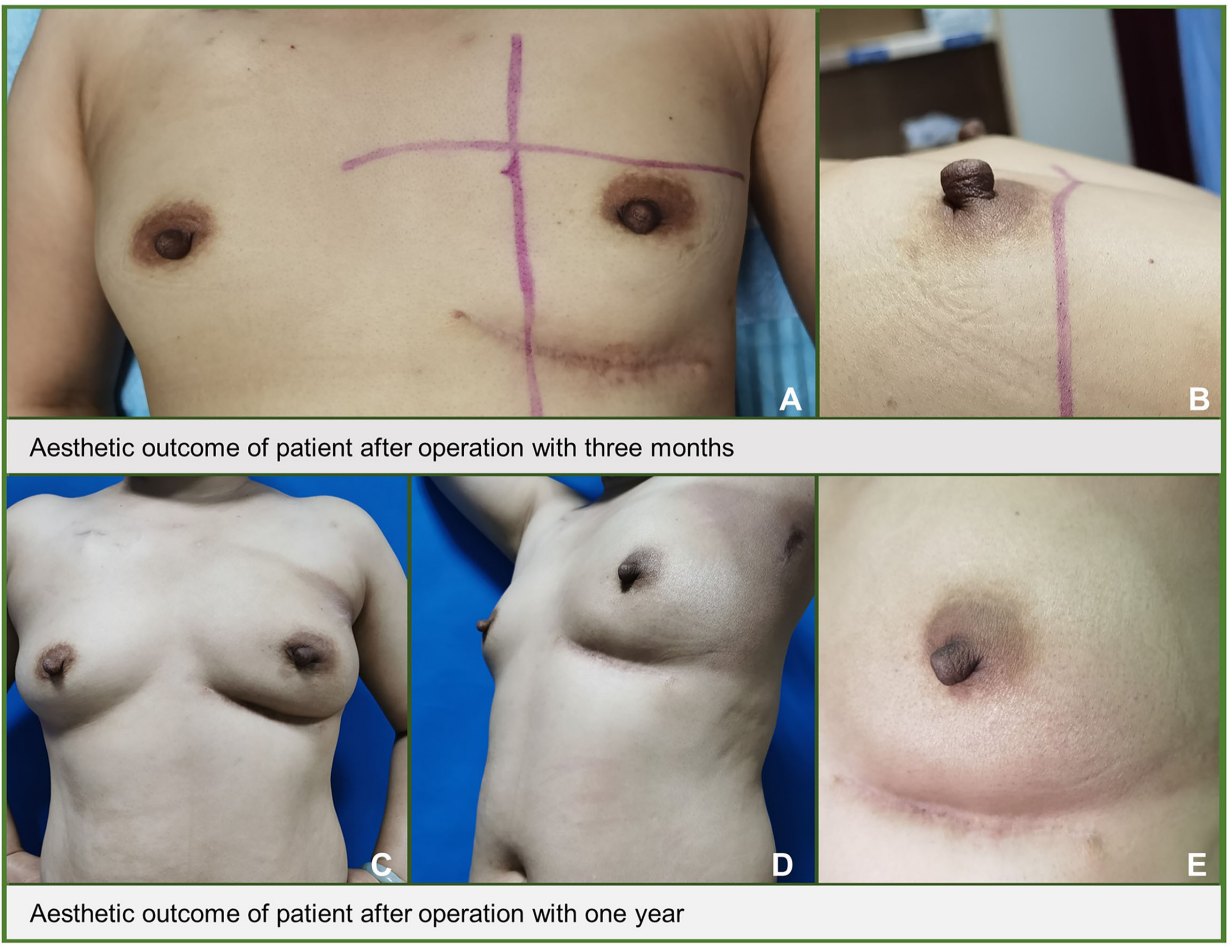
The aesthetic outcome of the breast after operation.

The mean aesthetic score was 4.7 points, with 36 patients scoring 5 points and 26 patients scoring 4 points, respectively (**Figures 4A, B**). **Figure 5** shows the follow-up data of patients at 3 months and 1 year after surgery. Overall, patients who underwent surgery with this technique were able to obtain a good aesthetic outcome without compromising their prognosis.

## Discussion

As previously reported by our research group, we offered an innovative, advantageous, and flexible oncoplastic technique to patients with small- to medium-volume breasts presenting with a tumor in the lower inner quadrant. The surgical approach allows sufficient glandular tissue transposition for reconstruction of the defect after complete tumor removal (10). Given our experience and other research reports, in the present study, we proposed the concept of the folding flap technique. This technique involves folding the prepared adjacent tissue flap directly into the postoperative residual cavity, making it a user-friendly procedure that provides adequate glands for reshaping the natural shape of the breast and distinguishing it from traditional flap rotation and transference.

Intercostal artery perforator (ICAP) flaps are a kind of commonly adjacent tissue flap collected from the body surface projection of the ICAP that are mainly used to repair defects in the lower quadrant and central area of the breast. It is not favorable to use ICAP flaps to repair defects in the inner and lower quadrants of the breast. In contrast, our incision design is simple and flexible and can be used to repair defects in the entire lower quadrant. In addition, unlike ICAP flaps, our flap is folded into the residual cavity and covered by the breast skin and subcutaneous fat, while a chromatic difference may exist between an ICAP flap and the surrounding skin, which can affect the visual appearance of the breast (11).

The thoracodorsal artery perforator (TDAP) flap, another commonly used adjacent tissue flap, also plays an important role in the repair of defects in the lower quadrant of the breast. This flap was developed from the latissimus dorsi muscle (LDM) flap. Because the LDM is preserved, this flap is effective in reducing postoperative shoulder disorders and the incidence of postoperative seroma (12). However, unlike our direct flap-folding technique, the TDAP flap technique requires that the flap be placed through a subcutaneous tunnel between the LDM and the defect area, which is a more complicated procedure than our technique requires. In addition, during flap transfer, the penetrating vessels, which are the main blood supply vessels, can be easily damaged by strain, increasing the risk of flap necrosis. In contrast, the blood supply to our flap comes mainly from the multiple intercostal perforator vessels surrounding the breast; thus, the operation procedure does not involve the major nerves and vessels, which means there is a lower probability of flap necrosis.

Another commonly used adjacent tissue flap is the inframammary adipofascial flap, which assists in the repair of defects in the lower quadrant of the breast using a tongue-shaped flap in the upper abdomen (13). This technique is consistent with our concept of incisional design. However, the flap in this technique is obtained primarily by freeing the subcutaneous fat and anterior rectus sheath from above to down through an IMF incision. This operation is limited by the length of the IMF incision, which makes it difficult to expose the surgical area and increases the difficulty of flap acquisition. Our flap is located on the surface of the upper abdomen and is, therefore, easier to obtain, requiring only a de-epithelialization of the previously designed semilunar area to prepare the appropriate flap.

The incision design is an important component in evaluating the postoperative aesthetic outcome of the breast. At present, as the classic incision designs for tumors in the lower quadrant of the breast, V and J shapes are widely used in clinical practice (14, 15). However, compared to our concealed inframammary crease incision, both of these incisions unavoidably leave a surgical scar on the breast surface after surgery, which affects the cosmetic appearance of the breast. On the other hand, our designed incisions are also covered by the lower pole of the breast under gravity after surgery and cannot be detected on the body surface. In addition, since the V-shaped incision benefits patients mainly with large-volume breasts having adequate glands, when applied to patients with small- to medium-volume breasts having insufficient glands, this incision design protocol increases the patient’s risk of postoperative breast deformity. In contrast, our incision design, which focuses on obtaining a flap around the breast, fills in the postoperative defect area and is not dependent upon the amount of self-generated glandular tissue available in the breast. Therefore, it is very friendly toward small- to medium-volume breasts. In addition, a J-shaped incision often leads to a localized bird-beak deformity of the breast while creating an ugly appearance of the breast (4). Our operation is performed in the posterior space of the breast and does not involve the surface of the breast, thus avoiding such deformities.

The occurrence of postoperative complications not only leads to compromised aesthetic results but also delays in postoperative adjuvant therapy (16). According to the report by Ho et al., among 30 patients who accepted the OPS technique, the postoperative complication rate was 21% (17), which is higher than our result of 13.5%. The possible reason for this discrepancy was the difference in flap source. In their study, Ho et al. used thoracoepigastric flaps and lateral intercostal artery perforator flaps as their primary flaps, which are both difficult to obtain and require a long operation, increasing the risk of postoperative complications. In relative terms, our flap can be more easily obtained, without extensive freeing of the gland, thus reducing the incidence of postoperative complications. Moreover, consistent with other studies, fat liquefaction was also the most common postoperative complication recorded among patients in our investigation. Research suggests that this complication may be related to thermal injury, diabetes, or obesity (18, 19). However, we did not include diabetic patients in our cohort, and not all of our cases with fat liquefaction had a high body mass index. Therefore, in this study, we did not consider these 2 risk factors as a priority when analyzing the causes of fat liquefaction in patients with incisions. Considering that the flap was freed by using an electro-knife, we suggest that thermal injury may be a primary risk factor for increased incisional fat liquefaction, but more clinical evidence is needed to confirm this hypothesis.

Oncoplastic surgery is performed to ensure tumor safety while maintaining the aesthetic outcome of the breast. Therefore, tumor safety is a prerequisite for the implementation of this technique. Here, we report a local recurrence rate of 3.8% (2/52) after a mean follow-up period of 28.5 months using the folding flap technique, which is significantly lower than that reported by other investigators (5%) (20). This finding suggests that our technique is safe when applied to patients; nevertheless, our result does not mean another technique is unsafe. Instead, the reason for such a difference may be due to the continuous updating of systemic treatment protocols. In addition, we also analyzed the causes of recurrence in 2 cases and found that the first patient with recurrence did not undergo postoperative radiotherapy. The absence of radiotherapy after breast-conserving surgery is believed to be strongly associated with the local recurrence of disease in 1 patient (21). A previous study demonstrated that young age was an independent risk factor for postoperative recurrence in breast cancer patients (22). This conclusion gives us a possible explanation for why our second case experienced recurrence after receiving systemic therapy, as she was only 33 years old.

Importantly, the limitations of our study cannot be ignored. First, the number of patients enrolled was limited; if possible, our technique needs to be validated in more centers and during a larger cohort study. Second, this technique might not be appropriate for very tin patients who have no bulk of tissue in the upper abdomen. In addition, longer follow-up is necessary to evaluate the tumor safety of our innovative technique, even though our short-term follow-up results suggest a better prognosis for patients.

## Conclusion

Our study offers an innovative and favorable folding flap technique for treating small- to medium-volume breasts with the tumor located in the lower quadrant. This technique could provide sufficient tissue to fill the residual cavity after the oncological operation while improving the aesthetic outcome of the breast. Thus, it may become an important complement of the mammary oncoplastic atlas.

## Data Availability Statement

The raw data supporting the conclusions of this article will be made available by the authors, without undue reservation.

## Ethics Statement

This study was approved by the medical ethics committee of the EUSOMA Certified Breast Center, Guilin TCM Hospital of China (Ethical code: GTCMH-2020-101; 26.08.2021) and the medical ethics committee of the Carl von Ossietzky University Oldenburg (Ethical code: 2021-139; 09.12.2021). The patients/participants provided their written informed consent to participate in this study. Written informed consent was obtained from the individual(s) for the publication of any potentially identifiable images or data included in this article.

## Author Contributions

RW and RZ have involved in the conception and design of the study. ZG, JD, and JZ collect the data. XS, ZTC, and ZHC analyzed the data. WS, MW, and LT-dR drafted the manuscript and figures. RW, RZ, and KW reviewed and edited the manuscript. All authors have read and approved the final manuscript.

## Funding

This study was supported by the University Medicine Oldenburg, Carl von Ossietzky University Oldenburg.

## Conflict of Interest

The authors declare that the research was conducted in the absence of any commercial or financial relationships that could be construed as a potential conflict of interest.

## Publisher’s Note

All claims expressed in this article are solely those of the authors and do not necessarily represent those of their affiliated organizations, or those of the publisher, the editors and the reviewers. Any product that may be evaluated in this article, or claim that may be made by its manufacturer, is not guaranteed or endorsed by the publisher.
