# Supplementary figures and images for: An Innovative Breast-Conserving Oncoplastic Technique for Treating Small to Medium Volume Breasts With a Tumor in the Lower Quadrant: The Folding Flap Technique
Source: Front Oncol. 2022 Mar 4;12:841682. doi: 10.3389/fonc.2022.841682 (PMC8931390; doi:10.3389/fonc.2022.841682)

**Supplement Figure 1:** Alternative method for folding flap technique.
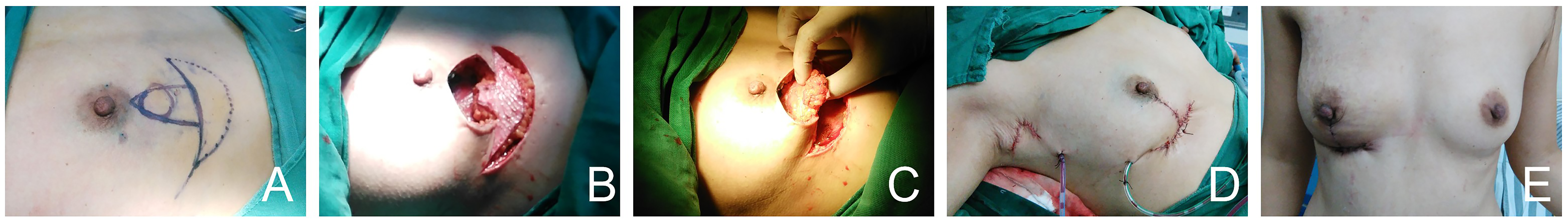

Supplement: Supplementary file 1 [file DataSheet_1.docx]
